# Correction: The Big Five personality traits and the fear of COVID-19 in predicting depression and anxiety among Japanese nurses caring for COVID-19 patients: A cross-sectional study in Wakayama prefecture

**DOI:** 10.1371/journal.pone.0315615

**Published:** 2024-12-05

**Authors:** The PLOS ONE Staff

There are errors in the author affiliations. The correct affiliations are as follows:

Ryo Odachi^1^, Shun Takahashi^2,3,4,5^, Daichi Sugawara^6^, Michiyo Tabata^3^, Tomomi Kajiwara^1^, Masaya Hironishi^7^, Momoko Buyo^1^

**1** Division of Health Sciences, Graduate School of Medicine, Osaka University, Suita City, Osaka, Japan, **2** Clinical Research and Education Center, Asakayama General Hospital, Sakai City, Osaka, Japan, **3** Department of Neuropsychiatry, Wakayama Medical University, Wakayama City, Wakayama, Japan, **4** Graduate School of Rehabilitation Science, Osaka Metropolitan University, Habikino City, Osaka, Japan, **5** Department of Psychiatry, Graduate School of Medicine, Osaka University, Suita City, Osaka, Japan, **6** Faculty of Human Sciences, University of Tsukuba, Tsukuba City, Ibaraki, Japan, **7** Wakyamma Medical University Kihoku Hospital, Ito Gun, Wakayama, Japan.

The publisher apologizes for the error.
