# Supplementary material for: The Big Five personality traits and the fear of COVID-19 in predicting depression and anxiety among Japanese nurses caring for COVID-19 patients: A cross-sectional study in Wakayama prefecture
Source: PLoS One. 2022 Oct 27;17(10):e0276803. doi: 10.1371/journal.pone.0276803 (PMC9612447; doi:10.1371/journal.pone.0276803)
Supplement: Attachment — Submitted filename: Response to Reviewers.doc [file pone.0276803.s002.doc]

We would like to express our deepest gratitude to the editor and reviewers. We have revised our manuscript to incorporate your suggestions.

Journal Requirements:

**1. When submitting your revision, we need you to address these additional requirements. Please ensure that your manuscript meets PLOS ONE's style requirements, including those for file naming.**

Response 1: Thank you for your comment. We have rechecked the journal’s style and modified the manuscript, including the references, accordingly. Additionally, we have opted for an English editing service to enhance the quality of the manuscript, especially focusing on grammar, structure, and the organization of writing.

**2. We note that the grant information you provided in the ‘Funding Information’ and ‘Financial Disclosure’ sections do not match. When you resubmit, please ensure that you provide the correct grant numbers for the awards you received for your study in the ‘Funding Information’ section.**

Response 2: Thank you for your comment. We have added the grant number in the “Funding Information” section.

**3. In your Data Availability statement, you have not specified where the minimal data set underlying the results described in your manuscript can be found. PLOS defines a study's minimal data set as the underlying data used to reach the conclusions drawn in the manuscript and any additional data required to replicate the reported study findings in their entirety. All PLOS journals require that the minimal data set be made fully available. Upon re-submitting your revised manuscript, please upload your study’s minimal underlying data set as either Supporting Information files or to a stable, public repository and include the relevant URLs, DOIs, or accession numbers within your revised cover letter. For a list of acceptable repositories, please see http://journals.plos.org/plosone/s/data-availability#loc-recommended-repositories. Any potentially identifying patient information must be fully anonymized. We will update your Data Availability statement to reflect the information you provide in your cover letter.**

Response 3: Thank you for your comment. We have thoroughly reviewed the ethical contents of the approved research plan and checked the availability of data. Finally, we have concluded that we can upload the minimal data set in a Supporting Information file. This has been described in the cover letter.

Additional Editor Comments:

Thank you for your instructive comments and suggestions. We have revised the manuscript in adherence to your advice.

Abstract:

**• Authors mentioned that “Our results showed an association between the HADS score and fear of COVID-19 and personality traits”- please consider mentioning whether this association is positive or negative.**

Response: Thank you for your comment. Following Reviewer 1’s recommendation, we have conducted a logistic regression analysis instead of a liner regression analysis. Therefore, some of the results presented have also changed in the revised manuscript. We have attempted to describe the results concretely and clearly. In the abstract, we have reported the odd rations with 95% confident intervals as well as the values of R squared.

**• Also, authors may consider mentioning the beta estimates with CI of multiple regression model.**

Response: Thank you for your comment. We have conducted a logistic regression analysis instead of a multiple regression analysis. Therefore, we have mentioned the odd rations and its CI in the revised abstract.

**Introduction:**

**• Citations are missing in several statements in the introduction. Please provide citations on all the facts.**

Response: Thank you for your comment. We have added the references for all such statements .

**• Fig 1 is blur, therefore losses readability.**

Response: Thank you for your comment. We have reworked on Fig. 1. Although it still appears to be blurred in the PDF, you can download the original version of Fig 1. from the link.

<https://www.dropbox.com/s/y1zgg1g7w26ry83/Figure1.tiff?dl=0>

Kindly provide suggestions regarding how we can present high-quality figures in the PDF as well.

**Methods:**

**• What is the study design?**

Response: Thank you for your comment. We have mentioned that this is a cross-sectional study in the first part of the “Methods” section.

**• Please provide inclusion and exclusion criteria.**

Response: Thank you for your comment. We have added the inclusion and exclusion criteria in the “Participants and procedures” section.

**• This study was approved by the Research Ethics Committee of Z University (approval no. zzzz). Does this University exist? The ref. number does not look real.**

Response: Thank you for your comment. We apologize for not presenting the name of the Research Ethics Committee correctly. We misunderstood the instructions regarding anonymized peer review and did not provide the name of the ethics committee in the manuscript. We have now presented the appropriate name and approval number of the committee.

**• Please provide the Cronbach alpha of all measures used in this study.**

Response: Thank you for your comment. As per your suggestion, we have added the Cronbach’s alpha of all the measures used in our study.

**Results:**

**• Please split the table 3 into two separate table since it has two different outcomes.**

Response: Thank you for your comment. As per your suggestion, we have modified the table to present the different outcomes. Additionally, we have conducted a logistic regression analysis instead of a multiple regression analysis, following Reviewer 1’s advice; the results of the logistic regression analysis are provided in Table 3.

**• Authors may not require repeating the results what was shown in the tables. I’d suggest just writing the key results presented in the table.**

Response: Thank you for your comment. As per your recommendation, we have modified the tables to present only the key results.

**• I’m confused to understand the variables when I look at the table 1 and table 2. I see that authors presented categorical anxiety and depression scale in table 1 and continuous value in table 2. Please clarify.**

Response: Thank you for your comment. We apologize for the lack of explanation on HADS. The HADS has a cut-off point for each subscale, depression, and anxiety. In Table 1, ‘Depression’ and ‘Anxiety’ presents each of the calculated HADS scores, while ‘Score of depression/anxiety’ indicates the number of people with depression/anxiety symptoms and people without symptoms. In Table 2, ‘Depression’ and ‘Anxiety’ represents the score of HADS. To avoid confusion, we have described the cut-off point of HADS.

**Discussion:**

**• Authors need to elaborately discuss the study findings including clearly stating the interpretation of the results and what was found in the earlier studies.**

**• Furthermore, discussion is warranted based on theoretical aspect.**

Response: Thank you for your instructive comments. We have revised the discussion section entirely to explain the findings of our study. In particular, we have focused on the relationship between FCV-19S, the Big Five personality traits, and depression/anxiety in our discussion. We have also compared the findings with those of previous studies.

**• I’d not use ‘mental health’ in the discussion rather use anxiety and depression.**

Response: Thank you for your comment. We agree with your suggestion. To express clearly, we have modified the term, “mental health” to “anxiety and depression.”

**• There are quite a number of limitations that authors should mention. For instance, generalizability, limitations of using self-reported data, causality...potential biases etc.**

Response: Thank you for your comment. We have added the limitations of the study such as those related to the measures used, self-reported questionnaires, procedure to distribute them, and sampling bias.

**Conclusion:**

**• Authors mentioned that “The results of this study indicated that the fear of COVID-19 and the Big Five personality traits could predict the levels of depression and anxiety using the HADS.” – I’m not sure what this means?**

**o I understand the fear of covid-19 increases anxiety and depression among the Nurses? Is this correct? If yes, please provide a clear recommendation rather than using ambiguous terms.**

**o I’m lost about the conclusion on the Big Five personality traits. I am not sure what authors are trying to say? Is it that those 5 personality traits predict increased anxiety and depression, sorry if I got it wrong? Please help me understand the conclusion.**

Response: Thank you for your comment. We apologize that our explanations in the “Conclusion” section were not clear. The findings of our study presented neuroticism to be a common risk factor of depression and anxiety in this revised manuscript. Additionally, the FCV-19S score was considered a risk factor only for anxiety. We have modified the “Conclusion” section to describe these results clearly.

Reviewers' comments:

**Reviewer #1**

Thank you for your careful review, instructive comments, and suggestions. We have revised the manuscript to incorporate your suggestions.

**- The abstract contains excessive information on the method (questionnaires distributed, answers received, ...), only the final number of participants should be indicated since the rest of the information is already exposed in the manuscript. Instead, the implications and/or interest of the results obtained should be added.**

Response: Thank you for your comment. Following your advice, we have modified the abstract to present less information on the methods used and added more information on the results of the logistic regression analysis.

**- The use of the keyword “mental health”, even in the title, is too broad. With it, it is expected to find the evaluation of symptomatology corresponding to more disorders, but the manuscript is limited to exploring depression and anxiety. It is recommended to specify the symptomatology studied.**

Response: Thank you for your comment. We agree with your suggestion. To express clearly, we have modified “mental health” to “anxiety and depression.” Furthermore, we have modified the title to “The role of Big Five personality traits and the fear of COVID-19 predict depression and anxiety among Japanese nurses caring for COVID-19 patients: A cross-sectional study in Wakayama prefecture.”

**- The authors comment that in Wakayama prefecture the incidence of Covid-19 is low and detail the third wave situation at the time of collecting information (page 9, lines 110-121), this information can be reduced without losing the content essential.**

Response: Thank you for your comment. As per your suggestion, we have reduced the number of descriptions explaining the situation at the time this study was conducted.

**- The use of Big Five personality, although widespread in the international community, is based on lexical aspects. There are other questionnaires with a robust psychobiological basis that are more suitable in the field of mental health work. Among them, Zuckerman's ZKPQ and Cloninger's TCI-R stand out. In the limitations section, this limitation should be added with the inclusion of the paper by Muro et al. (Chronobiology International, 28 (7), 690-696) in normal population and from Rio-Martinez et al. (Journal of Clinical Medicine, 9(6): 1876) in patients with mental disorders, the reading of both works in relation to the traits of personality of both questionnaires will provide the authors with this vision. The interest of considering more aspects of temperament or character, in the case of the TCI-R, is of the utmost importance. Introducing this approach also makes it possible to criticize the fact that the use of the Big Five personality presents relationships that are nonspecific in relation to the symptomatology studied.**

Response: Thank you for your suggestions. These articles were extremely helpful for us. As a result, we realized that there exists a limitation in the Big Five models. We understood your suggestion that the findings we have presented could be theoretically explained from the two domains of Temperament and Character and our results might be supported by the psychobiological basis if we used these. We have added this in the “Study limitation” section.

**- Measures. In this section, mention should not only be made of the reliability published in previous works, but also the detail of the reliability in the sample studied (FCV-19S, BFS and HADS) is required.**

Response: Thank you for your comment. Following your suggestion, we have added the information on the reliability and details of all scales, as discussed in previous studies.

**- In reference to the cut-off points of the HADS, the interest is focused on differentiating between “non-anxiety or depression” (0-7) and “possible case” (8 or more). Actually, the authors do not then work in their analyzes with the three possible groups indicated in the measures section (page 11). In table 1 it is not correct to define the depression and anxiety scores as low / high, I suggest adopting presence/absence of symptoms or yes/no possible case.**

Response: Thank you for your comment. A goal of our study is to screen and provide early support to nurses suffering from depression or anxiety. In this study, we attempt to find the possibilities of the occurrences of depression or anxiety. Therefore, it might not be appropriate to explain the cut-off points scoring with regard to the three groups in light of our purpose. Hence, we have modified the explanation regarding HADS; we used the cut-off points to divide the two groups of “non-anxiety or depression” and “possible case.” Additionally, we have modified the expression of the possible cases of depression or anxiety in Table 1, following your advice.

**- The choice of linear regression analysis is not the most appropriate. To establish the existence or not of an association between personality traits and symptoms of anxiety and depression (presence/absence or yes/no possible case), the approach requires logistic regressions. Since concluding that, for example, the more neuroticism the score is higher, it does not mean that a high neuroticism is really associated with scores for the presence of symptoms.**

Response: Thank you for your suggestion. Reconsidering the goal of our study, we realized that logistic regression analysis would be the more appropriate method for this study, as you had suggested. Consequently, we reanalyzed the data using this method and modified their related descriptions in the “Methods,” “Results,” “Discussion,” and “Conclusion” sections. According to the findings of this analysis, neuroticism was the only common risk factor of depression and anxiety while the FCV-19S score, age, and sex were risk factors only for anxiety.

**- In the results text (pages 14-15) the numerical data already provided in Table 1 are repeated. The two paragraphs should be rewritten in a more qualitative way and only highlighting some data of more interest.**

Response: Thank you for your comment. Following your suggestions, we have modified the results to present the critical data.

**- Presentation of data in the tables can be improved. In Table 2 the first line can be deleted and the results could be better interpreted if the full name of the variables is included. If you choose to keep the abbreviation, you must get the associated number and in the first line also indicate the abbreviation. In Table 3, if the p-values are presented, it is redundant and the column with asterisks should be deleted.**

Response: Thank you for your comment. As per your suggestion, we have revised Table 2; the first line was modified, and the full names of the variables were included.

**Reviewer #2: A good piece of work demonstrating the impact of psychological stressors on the front line nurses during the pandemic. It would be great if the authors and other researchers do further studies with other cultures and different healthcare settings.**

Response: Thank you for your comments and suggestions. Following the vaccination protocols, the COVID-19 situation in Japan is changing, and we will keep investigating this aspect further.
