# Supplementary material for: The Big Five personality traits and the fear of COVID-19 in predicting depression and anxiety among Japanese nurses caring for COVID-19 patients: A cross-sectional study in Wakayama prefecture
Source: PLoS One. 2022 Oct 27;17(10):e0276803. doi: 10.1371/journal.pone.0276803 (PMC9612447; doi:10.1371/journal.pone.0276803)
Supplement: Attachment — Submitted filename: Response to Reviewers.doc [file pone.0276803.s003.doc]

We would like to express our sincere gratitude to the editor and reviewers. We have revised our manuscript to incorporate your valuable suggestions.

Additional Editor Comments:

Thank you for your instructive comments and suggestions. We have now revised the manuscript as per your advice.

**(1) I am not sure I clearly understand the reason for using logistic regression. For instance, authors mentioned at the end of intro that "The purpose of this study was to clarify the relationship between the mental health problems of depression and anxiety in nurses who had to deal with patients afflicted with COVID-19 or was suspected COVID-19, the fear of COVID-19, and the Big Five personality traits." But based on the analysis, data nature and conclusion, it reads to me that authors investigated the risk/likelihood of depression and anxiety predicted by 5 traits, age and sex. Since the measures were collected as scale, I am not sure why authors considered to recode the cont. variable to cat. variable. I may have missed the point here.**

Response: Thank you for your comment. When we changed the method of the statistical analysis, we also changed the study’s purpose to investigate the risk of depression and anxiety in nurses, predicted by the fear of COVID-19 and the Big Five personality traits as described by you. It was because HADS was developed for screening depression and anxiety. Therefore, we thought it would be appropriate to analyze it as a categorical variable. This could show more clearly to what extent the scores of the Big Five scale and FCV-19S predict the risk for depression and anxiety. We noticed them due to the reviewer’s suggestions. We apologize for not changing the new purpose of the study. We have now modified the purpose appropriately to match the logistic regression analysis.

**(2) May I also kindly request authors to clarify why not 'linear mixed model with hospitals as random effect" is the best analysis approach for this study.**

Response: Thank you for your comment. As described above, we have changed the statistical method because we have considered that it would more explicitly present to readers the extent to which the independent variables affect nurses’ mental health problems. In the result of multiple linear regression, we thought it was a little difficult to understand how much of a problem the fluctuations in HADS scores brought by independent variables are regarded as clinical symptoms. We have added the reason to use logistic regression analysis in the “Statistical Analyses” section.

**(3) Regards to sex variable, I suggest authors to present the male and female in the table rather presenting as foot note the cat.**

Response 3: Thank you for your comment. As per your recommendation, we have now moved the label from the footnotes to the variable cells in Tables 3 and 4.

**(4) why sex is missing in the table 4.**

Response 4: Thank you for your comment. This was a simple mistake, and it seems that we have included it in another table that analyzed with this variable removed. We apologize for including the incorrect table. Thank you for pointing this out. Additionally, the values of the result of the logistic analysis in this manuscript text (lines 216-225) have been not modified because we had already referred to Table 4 in the previous manuscript.

**(5) Please consider providing some examples of each of the scales used in this study and state the response option. I see that authors mentioned that they have used 0-3 scale and what does the 0 represents.....**

Response 5: Thank you for your comment. We have added the explanation that high HADS scores mean more frequent symptoms or stronger symptoms. We have also included some examples of the question and the scoring method for its responses.

**(6) Please consider providing the scoring calculation of the score used in this study. For instance, authors mentioned that HADS has 7-items for depression. Is it the mean of 7 item or the total score treated as depression.**

Response 6: Thank you for your comment. The total score was derived adding up the scores of all the scales used in our study (HADS, FBS, FCV-19S). The total scores of each scale were used in the logistic regression analysis. We have added this in the explanations of each scale.

**(7) Please kind provide ref. of articles that used categorical HADS scale. Where does the cut-off for " is a score of more than 8" comes from.**

Response 7: Thank you for your comment. We apologize for the lack of reference. We have added the reference to explain the cut-off point.

**(8) Write full form of FCV19S in the tables.**

Response 8: Thank you for your comment. We have added the full form of FCV-19S in the footnotes of all the tables.

**(9) P8 L 119; what does “…...32% are aged" mean.**

Response 9: Thank you for your comment. In the sentence, it was meant that the aging rate of the population of Wakayama prefecture was 32%. We have now modified the explanation to be appropriate. We described this value as an indicator of the regional character of Wakayama prefecture that there is a relatively large older population because older age is considered a risk factor associated with disease severity.

**(10) English editing is necessary.**

Response 10: Thank you for your comment. We have opted for a professional English editing service to further enhance the quality of our entire manuscript.

Reviewers' comments:

**Reviewer #1**

Thank you for your careful review, instructive comments, and suggestions. We have revised the manuscript to incorporate your suggestions.

**- The presentation of data in tables can be improved. In Table 1 it is enough to detail the "yes". In Table 2 the line for the correlation between age and age should be deleted. In general, authors should carefully review the details of the tables.**

Response: Thank you for your suggestion. We have deleted this line from Table 2 as per your suggestion.

**Reviewer #2**

**- It seems the authors have tried to address the comments and enquiries of the reviewer accordingly and have made changes even to their methods. I think this work deserves being published.**

Response: Thank you for your supportive comments.
