# Supplementary material for: The Big Five personality traits and the fear of COVID-19 in predicting depression and anxiety among Japanese nurses caring for COVID-19 patients: A cross-sectional study in Wakayama prefecture
Source: PLoS One. 2022 Oct 27;17(10):e0276803. doi: 10.1371/journal.pone.0276803 (PMC9612447; doi:10.1371/journal.pone.0276803)
Supplement: Attachment — Submitted filename: Response to Reviewers.doc [file pone.0276803.s004.doc]

Dear Dr. Yunus,

Thank you for giving us the opportunity to submit a revised draft of our manuscript titled “The Big Five personality traits and the fear of COVID-19 in predicting depression and anxiety among Japanese nurses caring for COVID-19 patients: A cross-sectional study in Wakayama prefecture” to *PLOS ONE*. We appreciate the time and effort that you and the reviewers have dedicated to providing your valuable feedback on my manuscript. We are grateful to the reviewers for their insightful comments on my paper. We have been able to incorporate changes to reflect most of the suggestions provided by the reviewers. We have highlighted the changes within the manuscript.

Here is a point-by-point response to the reviewers’ comments and concerns.

Editor Comments:

**(1) The clarification for using multivariate logistic regression instead of mixed-effects logistic regression is not convincing. Please clarify the reason for not controlling random effect in the model A and Model B. Doesn’t hospital can be considered as a “random effect” variable which will account the variability of nurses’ experience across 13 hospitals? For example, isn’t it possible that nurses from a certain study hospital might experience more stress than others when exposed to the same level of stress.**

Response: Following your suggestion, we have added the explanation for performing multivariate logistic regression analysis below this sentence. Additionally, we have compared the scores of key scales (the HADS, the FCV-19S, and the BFS) between hospitals and have found no statistically significant differences. Therefore, it is considered that the variable “hospital” has a limited impact on the outcomes of this study. However, since the number of participants was unbalanced among the hospitals, it is possible that there might be differences in nurses’ experiences. We have added this in the limitations section.

Page 21, Lines 368–370

“*Additionally, the number of participants across hospitals was unbalanced, and it is possible that the differences in nurses’ experiences between hospitals were not reflected in these results*.”

**(2) Table 1: is it mean+/- SD or mean+/- SE. I think authors are using SD; however, I see in the table 3 and 4 authors used SE. Isn’t author should be consistent with SE across the paper.**

Response: Following your suggestion, we have modified our paper to use SE consistently. We have modified table 1 and parts of the method and results related to this.

**(3) P 14, L 216 authors wrote “Table 3 and Table 4 present the results of the multiple regression analysis for model A and B, respectively” isn’t should be multivariate logistic regression. I understand multiple regression represents linear regression.**

Response: We apologize for the error in the previous version of the manuscript. Following your feedback, we have modified it to “multivariate logistic regression.”

Page 14, Lines 224–225

*“Table 3 presents the results of the multivariate logistic regression analysis for models A and B*”

**(4) I think it might be useful to provide the VIF of the variables at the end of the table 2.**

Response: Although we agree with your suggestion, providing the VIF of the variables in Table 2 would make it too large. Therefore, we have added the VIFs in table 3, which presents the results of the logistic multivariate analysis.

**(5) It would be useful to merge table 3 and table 4 so that it can be easily understood by the reader.**

Response: Following your suggestion, we have merged Tables 3 and 4 into a new table, that is, Table 3.

**(6) First few lines of the discussion does not match with the revised study objective.**

Response: We have modified this sentence to match the study objective.

Page 16, Lines 240–241

“*This study aimed to assess the risk of depression and anxiety among nurses who have experience of caring for patients afflicted with COVID-19 in Japan.*”

**(7) Authors rightly cited previous studies in support to the study findings; however, I noticed that ‘Why” is missing throughout the discussion. Plus, how the study findings differed from the theory is essential to discuss.**

Response: Following your feedback, we noticed that we did not thoroughly explain the result that extraversion has no impact on depression and anxiety. Therefore, we have provided an explanation for this result in the discussion section.

Page 19, Lines 309–312

“*The pandemic has caused movement restrictions and people to avoid contact with each other. This may have brought about inadequate coping strategies for people with high extraversion. Therefore, it is considered that extraversion has not positively influenced nurses’ risk for depression and anxiety.*”
